# Therapeutic effect of TRC105 and decitabine combination in AML xenografts

**DOI:** 10.1016/j.heliyon.2020.e05242

**Published:** 2020-10-13

**Authors:** June Baik, Martin Felices, Ashley Yingst, Charles P. Theuer, Michael R. Verneris, Jeffrey S. Miller, Rita Perlingeiro

**Affiliations:** a Dept. of Medicine, University of Minnesota, Minneapolis, MN, USA; b Masonic Cancer Center, University of Minnesota, Minneapolis, MN, USA; c Dept. of Pediatrics, University of Denver, Colorado, CO, USA; d TRACON Pharmaceuticals, Inc., San Diego, CA, USA

**Keywords:** AML, TRC105, Decitabine, Xenografts, Cell biology, Antibody, Molecular biology, Cancer research, Chemotherapy, Hematology

## Abstract

Acute myeloid leukemia (AML) is an aggressive hematologic malignancy, often characterized by poor prognosis following standard induction therapy. The hypomethylating agent decitabine (DAC) is an alternative treatment for elderly and relapsed/refractory AML patients, yet responses following DAC monotherapy are still modest. The transforming growth factor-β (TGF-β) receptor CD105 (endoglin) is expressed in various hematopoietic malignancies, and high CD105 expression correlates with poor prognosis in AML patients. Using a xenograft model, we have recently demonstrated that targeting CD105^+^ AML blasts with the TRC105 monoclonal antibody inhibits leukemia progression. Here we investigated whether administration of TRC105 along with DAC could represent a novel therapeutic option for relapsed/refractory AML. Our data show that the DAC/TRC105 combination results in a more durable anti-leukemic effect in AML xenografts compared to DAC monotherapy. Moreover, the DAC/TRC105 combination enhanced reactive oxygen species (ROS) activity, which correlated with reduced leukemia burden. RNA-sequencing studies suggest that TRC105 may alter TGF-β activity in AML blasts. Taken together, these findings provide rationale for the clinical evaluation of TRC105 in combination with DAC in AML patients.

## 1 Introduction

Acute myeloid leukemia (AML) is the most common acute leukemia among adults, with a median onset age of 68 years (National Cancer Institute, 2012–2016). However, standard intensive chemotherapy aimed at achieving complete remission has not changed much in the past 4 decades [1]. Considering that 10–40% of elderly AML patients experience treatment-related mortality compared to less than 10% in younger patients, it is clear that the standard induction regimens are far from ideal for the elder patient population [2]. Furthermore, reduced-intensity conditioning often results in induction failure and consequently, less than 5% of elderly patients survive 5 years following the initial diagnosis [3]. Due to these limitations, treatment of elderly AML patients remains a significant challenge.

The hypomethylating agent decitabine (DAC) is considered a well-tolerated alternative treatment for AML patients [4]. DAC efficacy has been evaluated in both newly diagnosed and relapsed elderly AML patients, showing a 25–47% overall response rate as initial treatment [4]. Despite these promising outcomes, beneficial effects with DAC monotherapy require 3–4 cycles of treatment [1], and the survival rate in elderly AML patients remains poor (10–20% in 2016, National Cancer Institute). Therefore, there is an urgent need for novel therapeutic agents for the treatment of AML.

TRC105 is a human chimeric immunoglobulin G1 (IgG1) monoclonal antibody to CD105/endoglin, a receptor for the TGF-β superfamily. Due to the well-established role of CD105 in angiogenesis [5], TRC105 has been examined in phase 1–3 clinical trials for the treatment of advanced or metastatic solid cancers, which demonstrated safety [6]. Besides solid tumors, CD105 is also expressed in primary human leukemic blasts (36.9%) [7], and CD105 high expression correlates with poor prognosis in AML [8], suggesting that this receptor may also represent a potential target for hematopoietic malignancies. We have recently demonstrated that TRC105 administration to a human xenograft model of AML delays AML development and progression [9].

Based on the increasing application of DAC-based regimens as a salvage or frontline therapy for relapsed and unfit AML patients with suboptimal outcomes [4], here we investigated whether TRC105 in combination with DAC would provide enhanced therapeutic benefits. Our data show that a regimen in which leukemic mice receive DAC and TRC105 combined treatment results in reduced leukemia burden and elevated ROS activity of AML xenografts compared to DAC monotherapy. Moreover, our mechanistic studies provide molecular and cellular insights on TRC105 anti-leukemic activity, which is associated with TGFβ signaling, and independent of natural killer (NK) cells.

## 2 Results and discussion

**Figure 1:**
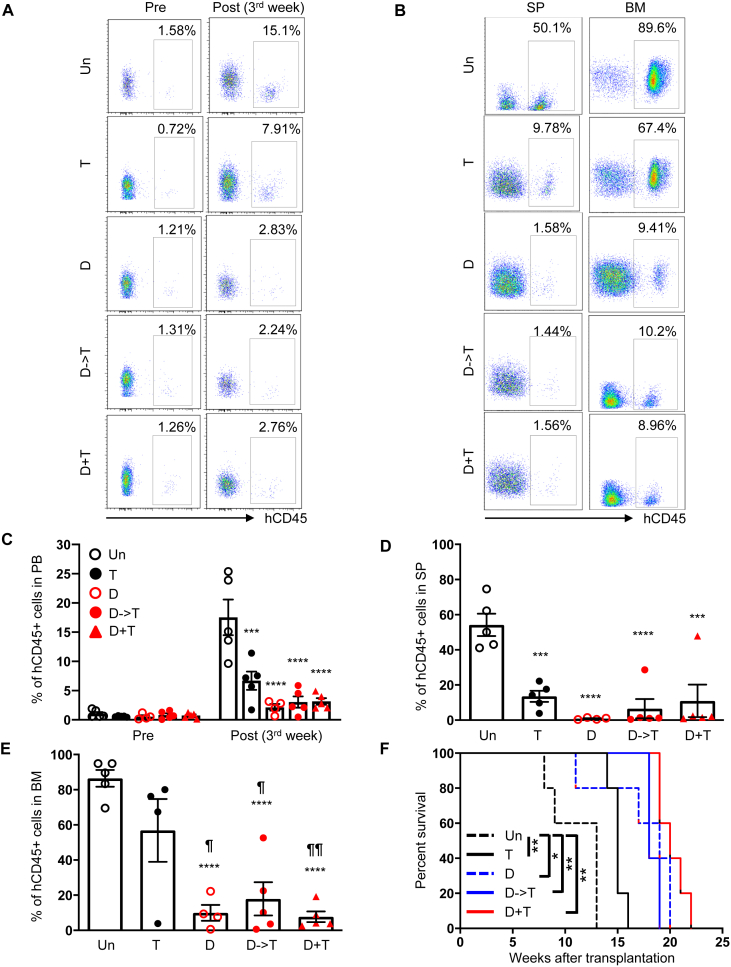
Effect of DAC and TRC105 in the AML xenografts after 3 weeks of treatment. (A) Representative FACS plots show the frequency of hCD45^+^ cells at pre- and post-treatment (3^rd^ week). (B) Representative FACS plots show expression levels of hCD45 in the SP (left) and BM (right) at the end of treatment (3^rd^ week). (C) Bars represent average frequency of circulating hCD45^+^ cells, and error bars represent standard error of the mean (SEM) for each cohort (n = 4–5 per group). ∗∗∗∗p < 0.0001 by ANOVA. (D–E) Graphs show the percentage of hCD45^+^ cells in the SP (D) and BM (E) upon completion of therapeutic regimen. Error bars represent SEM for each cohort. ¶p < 0.05, ¶¶p < 0.01, ∗∗∗p < 0.001, ∗∗∗∗p < 0.0001 by ANOVA. ¶ denotes comparisons to TRC105 monotherapy, and ∗ denotes comparisons to the untreated cohort. (F) Survival curve shows that concomitant administration of DAC and TRC105 provides the longest survival benefit among DAC-treated groups. BM from Figure 1B served as donor cells in secondary transplantation. The *x* axis represents weeks after secondary transplantation. ∗p < 0.05, ∗∗p < 0.01 by log-rank test.

We utilized a xenograft model to determine the therapeutic effect of DAC/TRC105 combination compared to DAC or TRC105 monotherapy for the treatment of AML. To better define the ideal regimen of DAC/TRC105 combinatorial treatment, TRC105 was administered either sequentially (post-DAC administration) or concomitantly with DAC. AML xenografts were established by infusing primary AML blasts into sub-lethally irradiated *NSG* (NOD.Cg-*Prkdc*^*scid*^ *Il2rg*^*tm1Wjl*^/SzJ) immunodeficient mice. Once the average frequency of human CD45-positive (hCD45^+^) cells in the peripheral blood (PB) was >1.5%, leukemic mice were divided into the following groups: i) untreated control, ii) TRC105 alone, iii) DAC followed by IgG isotype, iv) DAC followed by TRC105, and v) DAC and TRC105 concomitant administration. DAC was administered at 0.5 mg/kg daily for 5 consecutive days during the first week of treatment, as described in prior clinical studies [10]. Besides one cycle of DAC treatment, TRC105 or IgG isotype control was administered at 2 mg/kg using the intravenous route every 3 days until the end of treatment (3 or 8 weeks). Assessment at 3 weeks post-treatment show no significant differences between DAC alone and the DAC/TRC105 combination groups (Figure 1A-E), indicating that DAC/TRC105 combined regimen achieves comparable anti-leukemic effect to DAC monotherapy at short-term. To determine whether the DAC/TRC105 combination would ameliorate overall survival, we transplanted 5 × 10^6^ total bone marrow (BM) cells from the first cohort of xenografts into secondary recipients. To avoid bias, BM donors from DAC-treated primary transplants (D, D- > T, and D + T) were selected based on similar levels of hCD45^+^ (about 10%; Figure 1B). Even though there was no statistical difference among the recipients that had received BM from DAC-treated mice compared to DAC/TRC105 combinations, concomitant DAC/TRC105 treatment showed the longest survival among cohorts (Figure 1F), suggesting that DAC in combination with TRC105 may provide more durable responses.

**Figure 2:**
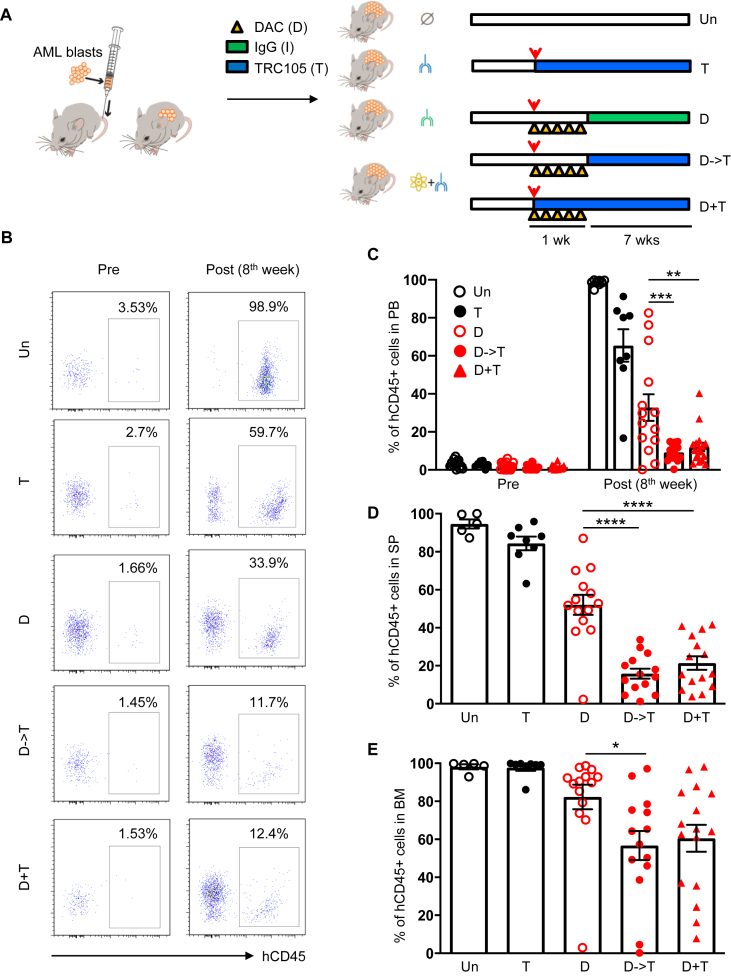
TRC105 enhances DAC anti-leukemic effect after 8 weeks of treatment. (A) Schematic outline of experimental design. Upon confirmation of the presence of hCD45^+^ cells in the PB, AML-bearing mice were divided into untreated (un), TRC105 monotherapy (T) and DAC-treated groups. In DAC-treated groups, mice received 5 doses of DAC daily followed by TRC105 (D- > T) or IgG isotype (control). Another cohort received concomitant administration of DAC and TRC105 (D + T). Red arrowheads indicate the onset of treatment. (B) Representative FACS plots show the frequency of hCD45^+^ cells in the PB before and after treatment. (C) Bars indicate average percentage of circulating hCD45^+^ cells. Error bars represent SEM for each cohort from two independent transplantation experiments (n = 10–18 mice per group). ∗∗∗p < 0.001 by ANOVA. (D–E) Frequency of hCD45^+^ cells in the SP (D) and BM (E) shows additive anti-leukemic effect of TRC105 and DAC combination. Error bars represent SEM for each cohort from two independent transplantation experiments (n = 5–16 mice per group). ∗p < 0.05, ∗∗∗<0.001, ∗∗∗∗p < 0.0001 by ANOVA.

**Figure 3:**
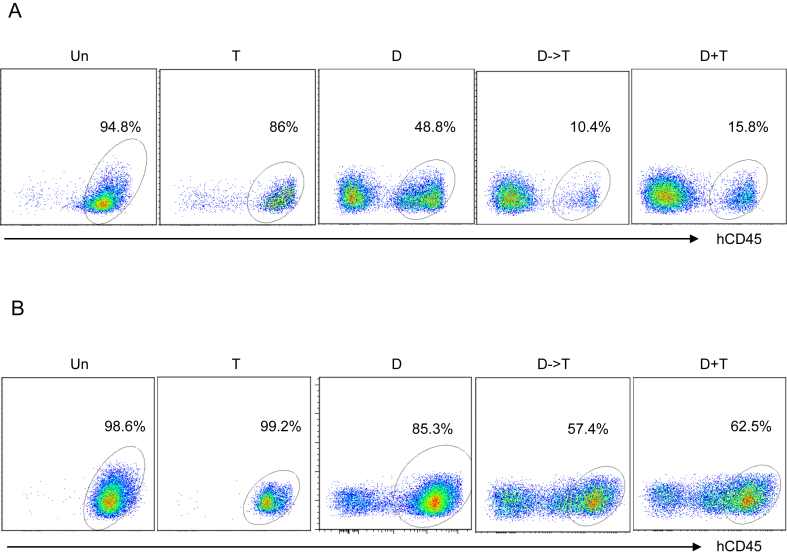
Effect of DAC and TRC105 in the BM and spleen of AML xenografts after 8 weeks of treatment. (A–B) Representative FACS plots show expression levels of hCD45 in the SP (A) and BM (B) at the end of treatment (8th week).

To determine whether the combinatorial treatment DAC/TRC105 may contribute to prolonged therapeutic effect in AML xenografts, we followed the experimental treatment described (Figure 2A), and assessed outcomes after 8 weeks. Once hCD45^+^ cells were detected in the circulation (Figure 2B-C, left), we started treatment regimens. Flow cytometry analysis of the PB at the end of treatment showed more than 95% hCD45^+^ cells in untreated mice, whereas xenografts receiving DAC monotherapy showed an average of 37% hCD45^+^ cells (Figure 2B-C). Remarkably, numbers of hCD45^+^ cells were the lowest (average 12%) in cohorts that had been treated with DAC/TRC105 combination (Figure 2B-C, right). Similar results observed in the spleen (SP) (Figure 2D and Figure 3A) and in the BM (Figure 2E and Figure 3B) of treated xenografts. Altogether, these data suggest that DAC/TRC105 combined therapy display more prolonged therapeutic effect in inhibiting leukemia burden compared to DAC alone in AML.

**Figure 4:**
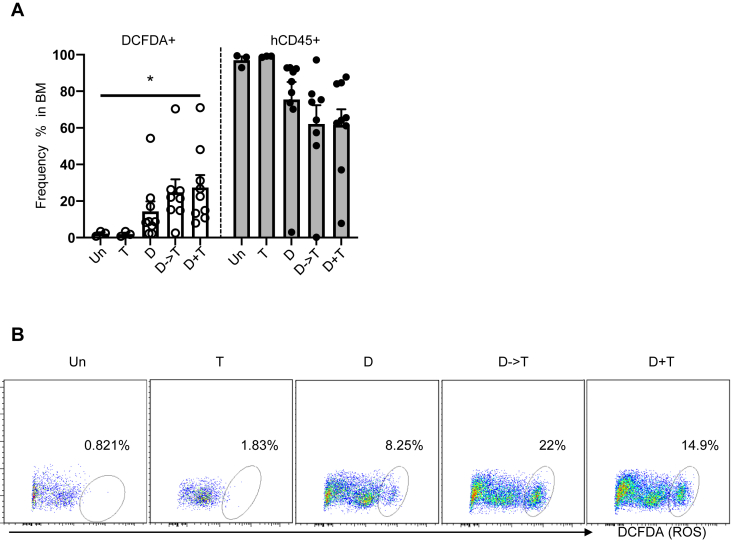
DAC/TRC105 combination enhanced ROS activity in AML xenografts. (A) Graph shows the percentage of DCFDA^+^ cells (left) and CD45^+^ cells in the BM (right) of untreated and treated cohorts (n = 3–9 per group). Higher frequency of DCFDA^+^ cells coincided with reduced leukemia burden (hCD45 positivity). ∗p < 0.05 by ANOVA. (B) Representative FACS plots show ROS activity in the BM of AML xenografts upon completion of therapeutic regimen. ROS activity is shown as frequency of DCFDA^+^ cells. Data show increased levels of ROS in AML xenografts treated with DAC/TRC105 combination.

Previous studies have shown that reactive oxygen species (ROS) interfere with the growth, survival, and genomic instability of leukemic blasts [11]. Azacitidine (AZA), a hypomethylating agent similar to DAC, in combination with the B-cell leukemia inhibitor venotoclax has recently provided promising clinical activity by modulating tumor metabolism through an increase in ROS levels [12]. To determine the impact of DAC/TRC105 treatment on leukemia metabolism, we examined intracellular ROS activity in the BM of these AML xenografts. Intracellular ROS activity was measured using 2′,7′-dichlorofluorescein diacetate (DCFDA) staining by flow cytometry. The lowest levels of ROS (<2.5%) were found in untreated and TRC105 monotherapy groups, whereas cohorts receiving the DAC/TRC105 combinatorial treatment displayed the highest percentages of ROS (Figure 4A, left and Figure 4B). Of note, this coincided with lessen leukemia burden as measured by levels of hCD45 (Figure 4A, right), suggesting that enhanced ROS activity by the combined treatment may contribute to therapeutic effect.

**Figure 5:**
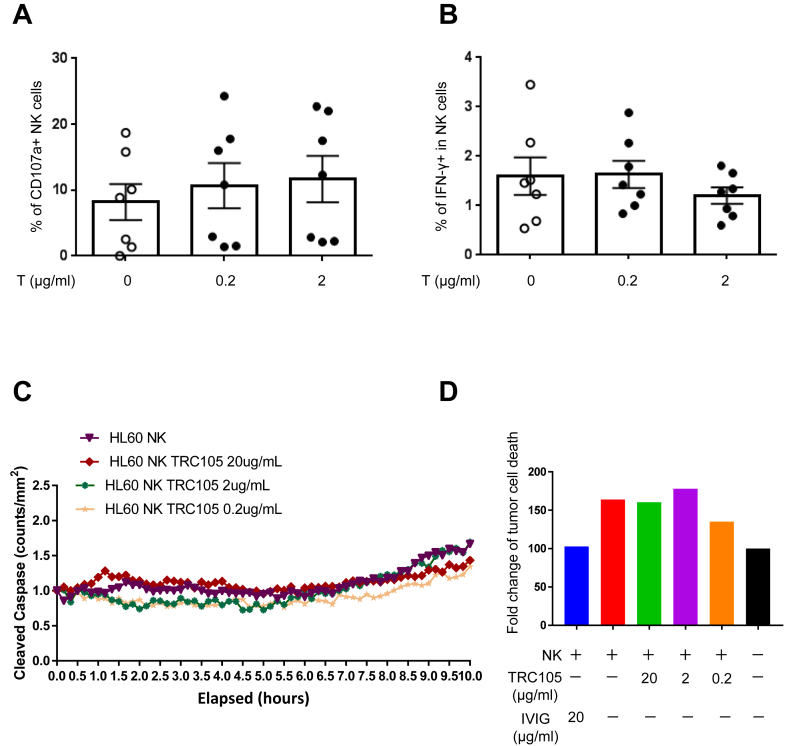
TRC105 anti-leukemic effect is independent of NK cells. Bars represent NK cell CD107a degranulation (A) and intracellular IFNγ production (B) on CD56+/CD3^-^ NK cells upon given concentration of TRC105. Error bars indicate SEM for each cohort from two independent experiments (n = 3–4 per experiment). (C) Shown are the results of HL60 cells cultured either alone or with freshly isolated NK cells (stimulated overnight with IL-2 at 50 U/ml) and increasing concentrations of TRC105. Experiments were performed in in triplicate at an E:T; 20:1 for 12 h and imaged by IncuCyte every 30 min. Cleaved caspase green was used as an indicator of HL60 apoptosis over time. (D) Fold change of tumor cell death, normalized to the amount of death when HL60 cells were grow in the absence of NK cells or TRC105 (black bars).

Therapeutic antibodies are currently in use to treat solid cancers and hematopoietic malignancies, including AML [13]. In most cases, the antitumor mechanism of therapeutic antibodies is based on antibody-dependent cellular cytotoxicity (ADCC) in which binding of these antibodies to tumor cells enable their recognition by immune effectors, such as natural killer (NK) cells [14]. To address this in a controlled *in vitro* setting, we evaluated the capacity of TRC105 to induce ADCC by testing human NK cells functional responses against the promyelocytic HL60 cell line, which abundantly expresses CD105 [9]. PB mononuclear cells (PBMC) were incubated with 0.2 and 2 μg/ml of TRC105 for 4 h in the presence of HL60. To assess NK cell activation, we measured NK cell degranulation via CD107a expression and intracellular IFNγ production [15]. No differences were observed in both these parameters between TRC105-treated and untreated groups (Figure 5A and 5B, respectively), demonstrating that TRC105 is unable to induce NK cell activation against CD105-expressing AML cells. Similarly, when HL60 cells were incubated with cord blood-derived NK cells, we did not observe TRC105-mediated increase in apoptosis, measured by amount of cleaved Caspase 3/7 in real time (Figure 5C-D). These findings demonstrate that NK cells are dispensable for TRC105-mediated anti-leukemic effect in AML.

**Figure 6:**
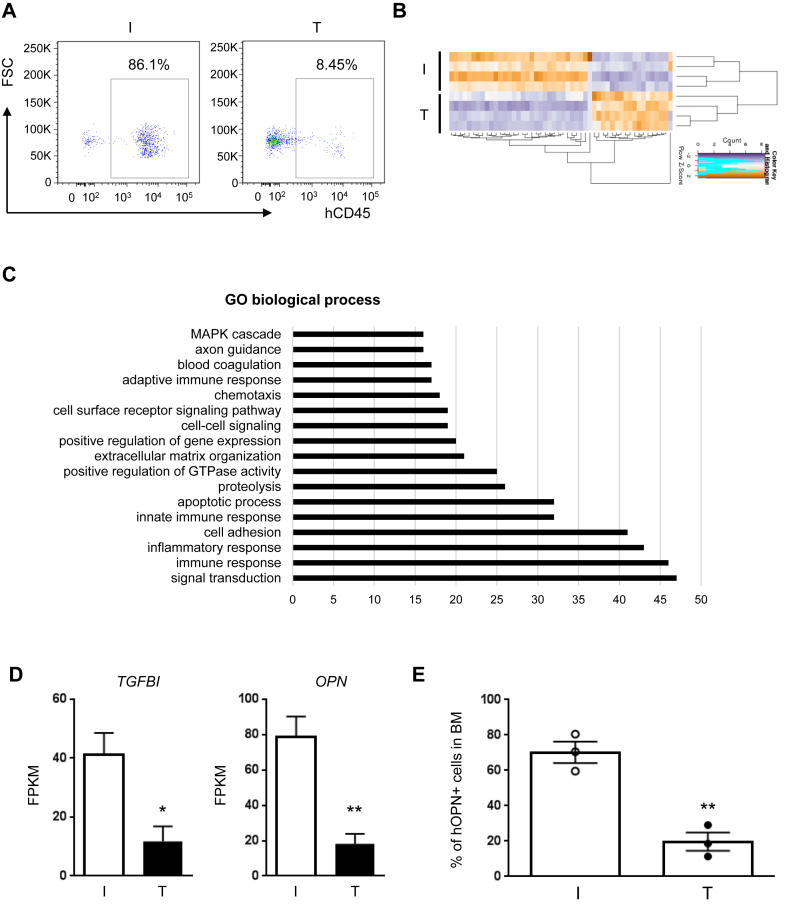
TRC105 inhibits TGF-β activity in AML blasts. (A) Representative FACS plots show levels of hCD45^+^ cells in the BM at 4 weeks post-injection. (B–D) RNA-seq analyses in AML blasts upon TRC105 treatment in xenografts. Two days after IV injection of 5 × 10^5^ AML blasts, *NSG* mice were randomly divided into two groups, TRC105 or IgG isotype control. (B) Heatmap represents the top 25 and bottom 25 variable genes in AML blasts between TRC105- and IgG-treated mice. (C) Gene ontology (GO) analysis of differentially regulated genes: TRC105 *vs.* IgG samples using DAVID. (D) Expression of the TGF-β regulated genes *TGFBI* and *OPN* in TRC105- and IgG-treated AML blasts. ∗p < 0.05, ∗∗p < 0.01 by t-test. (E) Bars represent the levels of human OPN^+^ cells in AML blasts from xenografts that had been treated with TRC105 or IgG isotype control. Error bars indicate SEM for each cohort (n = 3). ∗∗p < 0.01 by t-test.

Next, we performed whole-transcriptome analysis in AML blasts re-isolated from the BM of AML xenografts that had been treated with TRC105 or IgG isotype control for 4 weeks (Figure 6A). We identified 634 differentially expressed genes in the TRC105-treated cohort with respect to the IgG control group (Figure 6B). Gene ontology analysis of differentially expressed genes between TRC105- and IgG-treated AML blasts (Figure 6C) revealed several biological processes, including apoptosis, cell adhesion, and signal transduction. Among the significantly regulated genes, we observed that expression levels of *TGFBI* (Transforming Growth Factor Beta Induced) and *OPN* (Osteopontin) are significantly down-regulated in TRC105-treated AML blasts compared to IgG control counterparts (Figure 6D). TRC105-mediated down-regulation of OPN was further confirmed at the protein level by fluorescence activated cell sorting (FACS) in re-isolated BMs from recipients that had been treated with TRC105 compared to IgG controls (Figure 6E). Studies have reported TGF-β1-induced expression of *TGFBI* in several cancer cells [16], and a role for OPN in promoting tumor growth and metastasis through TGF-β signaling in breast cancer [17]. Of note, high expression of OPN correlates with poor overall survival in AML patients, supporting that OPN may represent a prognostic factor for AML [18, 19]. Given the well-established role of the TGF-β signaling pathway in tumor progression [20, 21, 22], our results suggest that TRC105 may repress leukemia progression by modulating TGF-β signaling in leukemic blasts. Nevertheless, based on the reported dichotomic role of TGF-β in tumor progression and malignant diseases [23, 24, 25], it is important to evaluate therapeutic outcomes associated with modulation of this pathway when testing novel regimens [26]. The results from this study indicate that TRC105-mediated modulation of TGF-β activity serves as a tumor-suppressor.

The survival rate for AML beyond 5 years of initial diagnosis is below 30% (National Cancer Institute, 2009–2015), and this drops to 5% in the elderly population [3]. Based on this urgent need, novel treatments have recently emerged to improve survival outcomes, including antibody therapies [27]. Of interest, TRC105 has been shown to be safe in clinical trials for the treatment of solid tumors, but to date, this antibody has not been examined as a therapeutic agent for AML. Here we show that TRC105 prolongs the anti-leukemic activity of DAC in AML xenografts, suggesting that TRC105 may provide beneficial effects to current treatment regimens for AML. While only DAC/TRC105 combination was examined in the present study, recent studies show that novel DAC nanocarrier has synergistic effects with chemotherapeutic reagents in various cancer models [28, 29, 30]. Further studies may be required to determine the most efficacious therapeutic combination, but our findings provide evidence for a novel combined regimen of DAC and TRC105 for AML, which may be particular benefit for AML patients who are unfit to standard induction therapy.

## 3 Methods

### 3.1 Primary samples

Primary human de-identified cryopreserved leukemic sample (AML-A0032), previously documented to give rise to leukemia upon injection into *NSG* mice [9], was obtained from the Hematology Malignancy Tissue Bank (HMTB) at the University of Minnesota, according to procedures approved by the Institutional Review Board of the University of Minnesota. AML-A0032 comes from a patient of CML transformed, with blast crisis, and characterized by (t(1; 3); del(5);-7; idem; t(1; 17)). Healthy human samples were obtained following informed consent, received in compliance with guidelines by the Committee on the Use of Human Subjects in Research and in accordance with the Declaration of Helsinki. Healthy donor blood was obtained from Memorial Blood Bank (Minneapolis, MN), and processed to isolate peripheral blood mononuclear cells (PBMCs) using density gradient Ficoll-Paque (GE Healthcare). PBMCs were cryopreserved in liquid nitrogen for later use.

### 3.2 Generation of Nuc-red expressing tumor lines

A 6-well plate was used to coat poly-ornithine (Sigma) and seeded with 1 × 10^6^ HL60 tumor cells. The plate was spun for 5 min at 500 xg. NucLight red lentivirus (Sartorius) was used at an MOI of 3 to transduce each tumor line. Cells were cocultured with virus for 24 h. After 24 h, media was replaced with fresh RPMI (Corning) with FBS (Seradigm) and penicillin-streptomycin (Gibco). After four days in culture, Nucred expressing cells were FACS sorted (using BD Aria) and expanded for use in the assays.

### 3.3 Mice and xenograft AML model

All experiments were approved by the University of Minnesota Institutional Animal Care and Use Committee. Female *NSG* (NOD.Cg-*Prkdc*^*scid*^ *Il2rg*^*tm1Wjl*^/SzJ; Jackson Laboratories) mice were 7–8 weeks of age at the time of transplantation. Sub-lethally irradiated (2.0 Gy) *NSG* mice were intravenously (IV) injected with 4–5 × 10^5^ primary AML-0032 cells. Mice were monitored weekly for signs of disease (including scruffy fur, weight loss, hunched posture and lethargy) and weekly for the presence of human CD45^+^ (hCD45^+^) cells in the peripheral blood (PB).

### 3.4 Treatment regimens of AML xenografts

TRC105 is an immunoglobulin G (IgG) monoclonal antibody specific for CD105 produced by TRACON Pharmaceuticals. TRC105 and human IgG (Thermo Fisher Scientific) antibodies were diluted in phosphate buffered saline (PBS, Gibco) prior to injection. DAC (5-Aza-2′-deoxycytidine, Sigma Aldrich) was dissolved in dimethyl sulfoxide at 2 mg/ml, as described [31], and further diluted to 0.5 mg/kg in PBS before injection. Treatment began 3 weeks after the injection of AML blasts, and experimental groups were divided as follows: (1) untreated, (2) TRC105 alone (2 mg/kg IV, every 3 days), (3) TRC105 and DAC (0.5 mg/kg IP, daily for 5 days) concomitant (D + T), (4) DAC followed by TRC105 administration (D- > T), and (5) DAC followed by IgG isotype control administration. TRC105 was continuously administered until the end of the study. For RNA-sequencing studies, TRC105 or IgG isotype control was administered from day 2 of AML injection to the end of the study.

### 3.5 Flow cytometry

To eliminate erythrocytes, PB, BM and SP samples were incubated with ACK lysing solution (Thermo Fisher Scientific) at room temperature for 10–20 min prior to antibody staining. PB, BM and SP cells were stained with PE-Cy7-conjugated human CD45 (clone HI30; BD Biosciences) on ice for 30 min. For osteopontin staining, BM cells were incubated in 250 μl of BD Cytofix/Cytoperm Fixation and Permeabilization Solution (BD Biosciences) for 20 min prior to eFluor 660-conjugated anti-Osteopontin antibody (clone 2F10, eBioscience). To measure the level of intracellular reactive oxygen species (ROS), we stained with 20μM 2′,7′-dichlorofluorescin diacetate (DCFDA) (ab1131851, Abcam) for 30 min at 37 °C. Flow cytometry analyses and sorting were performed using a FACSAria II cytometer (BD Biosciences). FlowJo software was used for analysis (Tree Star Inc.).

### 3.6 NK cell functional assays

PBMCs were thawed and rested in RPMI with 10% FBS overnight in a 37 °C incubator with 5% CO_2_ prior to use. NK cells for the IncuCyte Cytotoxicity Assay were isolated from healthy donor peripheral blood mononuclear cells via ficoll density gradient centrifugation and further isolated from PBMC's using the EasySep NK enrichment cocktail (STEMCELL Technologies) obtained from the University of Colorado Children's Hospital Blood Bank. NK cells were then cultured in NK media (B0) overnight in the presence of 50U/mL of IL-2 (R&D Systems) and used within 24 h for the ADCC assays.

### 3.7 CD107a degranulation and IFNγ cytokine production assay

PBMCs were plated overnight and then co-cultured for 4 h with HL-60 at an effector/target ratio of 2:1 with the noted treatments. FITC-conjugated anti-CD107a (clone H4A3; BioLegend), used to evaluate NK cell degranulation, was added at start of the 4-hour incubation period. After 1-hour incubation, Golgi Stop and Golgi Plug (BD Biosciences) were added for the last 3 h. At the end of the 4 h, the cells were stained using the Live/Dead Fixable Aqua Staining Kit (Thermo Fisher Scientific), surface stained with PE-Cy7-conjugated anti-56 (clone HCD56; BioLegend) and PE-CF594-conjugated anti-3D (clone 3G8; BioLegend), fixed with 2% paraformaldehyde, and permeabilized with intracellular staining buffer (BioLegend). The cells were stained for BV650-conjugated IFNγ (clone 4S.B3; BioLegend). NK cells were identified as CD56^+^/CD3^-^/live cells and gated into CD56 bright and CD56 dim fractions. Cells were run on LSRII (BD Biosciences) for 60 s per sample, analyzed with FlowJo software, and graphed on GraphPad Prism software.

### 3.8 IncuCyte Cytotoxicity Assay

An automated live-cell imaging system (IncuCyte Essen Bioscience) was used to evaluate NK cell cytotoxicity against CD105 expressing HL60 cells with and without the presence of TRC105 (Tracon Pharmaceuticals) or IVIG (control). Nuc-red HL60 tumor cells were plated atop poly-ornithine (sigma) coated wells at 2.5 × 10^3^ cells/100uL well in NK media (B0). Tumor cells were spun down for 5 min at 500xg. NK cells/well cells were added per well at a 1:20 effector:target ratio. TRC105 was added at either 0.2ug/mL, 2ug/mL, or 20ug/mL. Caspase green reagent (Sartorius) was added in at 5uM/well. Each well was topped with NK media to a total of 200uL/well. Controls included tumor cells alone, NK + tumor alone (no antibodies), NK + tumor + IVIG at 20ug/mL. Each condition was performed in triplicate. Cells were analyzed on the incucyte every 30 min for a total of 12 h under 10x magnification. Data was analyzed using incucyte software and graphpad prism. The detection of caspase green and nucred (tumor) was quantified over time. Data was normalized to tumor cells (100%) to represent fold change of tumor cell death using the following formula= (AUC of Caspase/AUC of nuc-red)/background x 100.

### 3.9 RNA sequencing

RNA was isolated from 50,000 hCD45^+^ cells that had been purified by FACS from the BM of AML-bearing mice, using the PicoPure RNA Isolation Kit (Thermo Fisher Scientific) following the manufacturer's instructions for “RNA Extraction from Cell Pellets” (including DNase Treatment). Total RNA (greater than 20 ng) from 4 independent replicates and 2 different cohorts (total 8 independent samples) were submitted to the University of Minnesota Genomic Center for library preparation and sequencing. Libraries were generated using the Clontech Smarter Stranded Total RNA Pico Input Mammalian kit (Takara Bio). All libraries were normalized, combined into a single pool, and sequenced on a HiSeq 2500 instrument operated at high output mode, 50 bp PE run, which generated around 10 million reads per sample. Paired-end reads were trimmed using Trimmomatic (v 0.33) enabled with the optional “-q” option; 3bp sliding-window trimming from 3′ end requiring minimum Q30. Quality control on raw sequence data for each sample was performed with FastQC. Read mapping was performed via Hisat2 (v2.0.2) using the human genome (hg19) as reference. Gene quantification was done via Cuffquant for FPKM values and Feature Counts for raw read counts. Differentially expressed genes were identified using the edgeR (negative binomial) feature in CLCGWB (Qiagen) using raw read counts. We filtered the generated list based on a minimum 2X Absolute Fold Change and FDR corrected p < 0.05. The R heatmap.3 function was used to display the resulting heatmap. Functional annotation of differentially expressed genes was performed using the online tool DAVID.

### 3.10 Statistical analysis

Differences between samples were determined by using the unpaired Student's *t*-test for single comparisons, or one-way ANOVA for multiple comparisons followed by the Dunnett test. Log-rank (Mantel–Cox) test was used to compare survival distributions. *p* values <0.05 were considered significant.

## Data availability

The RNA-Seq data are available at the Gene Expression Omnibus under accession number GSE147503.

## Declarations

### Author contribution statement

R. Perlingeiro: Conceived and designed the experiments; Analyzed and interpreted the data; Wrote the paper.

J. Baik: Conceived and designed the experiments; Performed the experiments; Analyzed and interpreted the data; Wrote the paper.

M. Felices and A. Yingst: Performed the experiments; Analyzed and interpreted the data.

C.P. Theuer: Contributed reagents, materials, analysis tools or data.

M.R. Verneris and J.S. Miller: Analyzed and interpreted the data.

### Funding statement

J.S. Miller was supported by 10.13039/100000002National Institutes of Health (P01 CA111412). J. Baik was supported by 10.13039/100000002National Institutes of Health (2T32HL007062). R. Perlingeiro was supported by TRACON Pharmaceutical and Masonic Cancer Center, 10.13039/100007249University of Minnesota.

### Competing interest statement

The authors declare the following conflict of interests: Author R. Perlingeiro has received research support from TRACON. Author C.P. Theuer is an employee of TRACON.

### Additional information

No additional information is available for this paper.

## References

[1] O'Donnell M.R., Tallman M.S., Abboud C.N. (2017). Acute myeloid leukemia, version 3.2017, NCCN clinical practice guidelines in oncology. J. Natl. Compr. Canc. Netw..

[2] Pollyea D.A., Kohrt H.E., Medeiros B.C. (2011). Acute myeloid leukaemia in the elderly: a review. Br. J. Haematol..

[3] Almeida A.M., Ramos F. (2016). Acute myeloid leukemia in the older adults. Leuk Res. Rep..

[4] Kantarjian H.M., Thomas X.G., Dmoszynska A. (2012). Multicenter, randomized, open-label, phase III trial of decitabine versus patient choice, with physician advice, of either supportive care or low-dose cytarabine for the treatment of older patients with newly diagnosed acute myeloid leukemia. J. Clin. Oncol..

[5] Nassiri F., Cusimano M.D., Scheithauer B.W. (2011). Endoglin (CD105): a review of its role in angiogenesis and tumor diagnosis, progression and therapy. Anticancer Res..

[6] Seon B.K., Haba A., Matsuno F. (2011). Endoglin-targeted cancer therapy. Curr. Drug Deliv..

[7] Cosimato V., Scalia G., Raia M. (2018). Surface endoglin (CD105) expression on acute leukemia blast cells: an extensive flow cytometry study of 1002 patients. Leuk. Lymphoma.

[8] Kauer J., Schwartz K., Tandler C. (2019). CD105 (Endoglin) as negative prognostic factor in AML. Sci. Rep..

[9] Dourado K.M.C., Baik J., Oliveira V.K.P. (2017). Endoglin: a novel target for therapeutic intervention in acute leukemias revealed in xenograft mouse models. Blood.

[10] Cashen A.F., Schiller G.J., O'Donnell M.R., DiPersio J.F. (2010). Multicenter, phase II study of decitabine for the first-line treatment of older patients with acute myeloid leukemia. J. Clin. Oncol..

[11] Irwin M.E., Rivera-Del Valle N., Chandra J. (2013). Redox control of leukemia: from molecular mechanisms to therapeutic opportunities. Antioxidants Redox Signal..

[12] Pollyea D.A., Stevens B.M., Jones C.L. (2018). Venetoclax with azacitidine disrupts energy metabolism and targets leukemia stem cells in patients with acute myeloid leukemia. Nat. Med..

[13] Majeti R. (2011). Monoclonal antibody therapy directed against human acute myeloid leukemia stem cells. Oncogene.

[14] Weiner L.M., Surana R., Wang S. (2010). Monoclonal antibodies: versatile platforms for cancer immunotherapy. Nat. Rev. Immunol..

[15] Vallera D.A., Felices M., McElmurry R. (2016). IL15 trispecific killer engagers (TriKE) make natural killer cells specific to CD33+ targets while also inducing persistence, in vivo expansion, and enhanced function. Clin. Canc. Res..

[16] Schneider D., Kleeff J., Berberat P.O. (2002). Induction and expression of betaig-h3 in pancreatic cancer cells. Biochim. Biophys. Acta.

[17] Weber C.E., Kothari A.N., Wai P.Y. (2015). Osteopontin mediates an MZF1-TGF-beta1-dependent transformation of mesenchymal stem cells into cancer-associated fibroblasts in breast cancer. Oncogene.

[18] Chen Y.B., Ren S.M., Li S.D., Du Z. (2017). Prognostic significance of osteopontin in acute myeloid leukemia: a meta-analysis. Mol. Clin. Oncol..

[19] Liersch R., Gerss J., Schliemann C. (2012). Osteopontin is a prognostic factor for survival of acute myeloid leukemia patients. Blood.

[20] Batlle E., Massague J. (2019). Transforming growth factor-beta signaling in immunity and cancer. Immunity.

[21] Tabe Y., Shi Y.X., Zeng Z. (2013). TGF-beta-Neutralizing antibody 1D11 enhances cytarabine-induced apoptosis in AML cells in the bone marrow microenvironment. PloS One.

[22] Seoane J., Gomis R.R. (2017). TGF-beta family signaling in tumor suppression and cancer progression. Cold Spring Harb. Perspect Biol..

[23] Fagone P., Mangano K., Pesce A., Portale T.R., Puleo S., Nicoletti F. (2016). Emerging therapeutic targets for the treatment of hepatic fibrosis. Drug Discov. Today.

[24] Kandasamy M., Anusuyadevi M., Aigner K.M. (2020). TGF-beta signaling: a therapeutic target to reinstate regenerative plasticity in vascular dementia?. Aging Dis..

[25] Petralia M.C., Mazzon E., Fagone P. (2020). The cytokine network in the pathogenesis of major depressive disorder. Close to translation?. Autoimmun. Rev..

[26] Mancarella S., Krol S., Crovace A. (2019). Validation of hepatocellular carcinoma experimental models for TGF-beta promoting tumor progression. Cancers (Basel).

[27] Williams B.A., Law A., Hunyadkurti J., Desilets S., Leyton J.V., Keating A. (2019). Antibody therapies for acute myeloid leukemia: unconjugated, toxin-conjugated, radio-conjugated and multivalent formats. J. Clin. Med..

[28] Li S.Y., Sun R., Wang H.X. (2015). Combination therapy with epigenetic-targeted and chemotherapeutic drugs delivered by nanoparticles to enhance the chemotherapy response and overcome resistance by breast cancer stem cells. J. Contr. Release.

[29] Briot T., Roger E., Bou Haidar N. (2019). Di-O-lauroyl-decitabine-lipid nanocapsules: toward extending decitabine activity. Int. J. Nanomed..

[30] Sahli F., Courcelle M., Palama T., Djaker N., Savarin P., Spadavecchia J. (2020). Temozolomide, gemcitabine, and decitabine hybrid nanoconjugates: from design to proof-of-concept (PoC) of synergies toward the understanding of drug impact on human glioblastoma cells. J. Med. Chem..

[31] Mangano K., Fagone P., Bendtzen K. (2014). Hypomethylating agent 5-aza-2'-deoxycytidine (DAC) ameliorates multiple sclerosis in mouse models. J. Cell. Physiol..

